# Risk Analysis Index Versus mFI‐5 for Predicting Outcomes After Uvulopalatopharyngoplasty

**DOI:** 10.1002/lary.70571

**Published:** 2026-04-16

**Authors:** Akshay Warrier, Ariana Shaari, Aman M. Patel, Christian Bowers, Kenneth Yan

**Affiliations:** ^1^ Department of Otolaryngology—Head and Neck Surgery Rutgers New Jersey Medical School Newark New Jersey USA; ^2^ Department of Otolaryngology Stony Brook University New York USA; ^3^ Bowers Frailty and Surgical Outcomes Lab Flint Michigan USA

**Keywords:** frailty, modified frailty index‐5, obstructive sleep apnea, postoperative complications, risk analysis index, surgical outcomes, uvulopalatopharyngoplasty

## Abstract

**Objective(s):**

Current preoperative risk stratification for uvulopalatopharyngoplasty (UPPP) primarily relies on anatomic and disease‐severity measures, which do not capture physiologic reserve or vulnerability to postoperative complications. This study aimed to compare the predictive performance of the risk analysis index (RAI) and the modified frailty index‐5 (mFI‐5) in stratifying postoperative risk among patients undergoing UPPP.

**Methods:**

Adult patients who underwent UPPP between 2005 and 2020 were identified. Frailty was assessed, and associations with postoperative complications, length of stay, discharge disposition, and mortality were evaluated. Multivariable regression and receiver operating characteristic analysis were conducted.

**Results:**

A total of 2129 patients were included and severe frailty (RAI > 31) was strongly associated with adverse postoperative outcomes, including Clavien–Dindo (CD) II complications (OR 21.3, 95% CI 3.7–123.8), CD IV (OR 4.6, 95% CI 2.0–10.6), extended length of stay (eLOS) (OR 29.4, 95% CI 5.4–160), non‐home discharge (NHD) (OR 6.1, 95% CI 3.0–12.6), and deep surgical site infection (DSSI) (OR 17.5, 95% CI 1.7–176.3) (all *p* < 0.05). The mFI‐5 predicted CD II, eLOS, and NHD but not CD IV or DSSI among severely frail patients (mFI‐5 > 3). RAI consistently outperformed mFI‐5 across outcomes, including mortality (AUC 0.813 vs. 0.580), CD II (0.784 vs. 0.652), CD IV (0.704 vs. 0.654), eLOS (0.767 vs. 0.669), and NHD (0.767 vs. 0.618).

**Conclusion:**

Although both RAI and mFI‐5 are associated with postoperative outcomes following UPPP, RAI provides superior discrimination for mortality and major morbidity. These findings support the incorporation of RAI into preoperative risk stratification for sleep surgery to better inform perioperative planning and resource allocation.

**Level of Evidence:**

3.

## Graphical abstract

This retrospective database study of 2129 adults undergoing uvulopalatopharyngoplasty compared the risk analysis index (RAI) with the modified 5‐item frailty index (mFI‐5) for predicting 30‐day postoperative outcomes and found that frailty was associated with worse morbidity, prolonged hospitalization, non‐home discharge, and mortality. Although both tools showed some predictive value, RAI demonstrated substantially stronger discrimination across key endpoints, including mortality, Clavien–Dindo II and IV complications, extended length of stay, non‐home discharge, and deep surgical site infection. These findings suggest that RAI is a more comprehensive and clinically useful frailty measure than mFI‐5 for preoperative risk stratification in UPPP patients. Incorporating RAI into sleep surgery workflows may improve perioperative planning, patient counseling, and resource allocation.

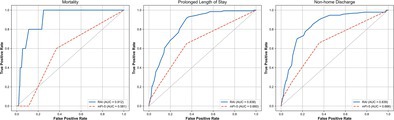

## 1 Introduction

Obstructive sleep apnea (OSA) is associated with significant morbidity and mortality due to its links with cardiovascular disease, metabolic dysfunction, and impaired quality of life, making surgical management an important consideration for patients who fail conservative therapies [1]. Uvulopalatopharyngoplasty (UPPP) was first introduced in 1979 for the treatment of OSA [2]. It involves the removal of the posterior soft palate and uvula, followed by modification of the anterior and posterior tonsillar pillars [3]. Although treatment paradigms for OSA continue to evolve, palatal surgery remains a cornerstone of multilevel surgical management, with UPPP performed in appropriately selected patients [4, 5]. Postoperative complications associated with UPPP include pain, dehydration, velopharyngeal insufficiency, dysphagia, hemorrhage, airway compromise, surgical site infection, and cardiopulmonary events, which may necessitate hospital readmission, surgical reintervention, or result in death [6]. Given the potential morbidity related to post‐surgical complications, it is important to identify risk factors associated with adverse outcomes to improve patient selection for UPPP, enhance patient education, and personalize perioperative treatment strategies. As the demand for surgical management increases, such risk‐stratification is crucial.

Frailty refers to a physiological syndrome of decreased physiological reserve that makes a patient vulnerable to poor postoperative outcomes [7]. While frailty has been identified as a predictor of poor postoperative outcomes in numerous surgical specialties, including otolaryngologic surgeries, no study has explored the use of frailty as a predictor for UPPP [7]. Additionally, there is no consensus among the numerous metrics that have been developed to quantify frailty [8, 9, 10, 11, 12, 13]. The modified 5‐item frailty index (mFI‐5) is one of the most ubiquitous frailty metrics, which is a composite metric of various comorbidities [14]. Another commonly used frailty index is the modified 11‐item frailty index (mFI‐11), which is similar to mFI‐5 but with 11 variables. Both mFI‐5 and mFI‐11 have been shown to be predictive of complications after surgery in surgical specialties including neurosurgery, orthopedic surgery, and general surgery [14, 15, 16, 17, 18, 19, 20, 21]. However, there are also reports on the limitations of mFI, including the poor predictive potential in some surgeries, and the potential oversimplification of the complex nature of frailty, as mFI primarily relies on comorbidity‐based variables and may fail to capture important domains such as cognitive, functional, and psychosocial status. The risk analysis index (RAI), a more recently developed metric that measures frailty across several domains such as cognitive function, physical health, and social support, has shown potential in several recent studies reporting on the superiority of RAI over mFI in predictive postoperative outcomes [22, 23, 24, 25]. The RAI assessments were designed to optimize preoperative screening in patients undergoing elective surgery, assessing frailty across multiple variables and domains. These include general patient demographics such as age and sex, medical comorbidities (heart failure, renal impairment, dyspnea, and cancer), overall nutrition (described as weight loss), and a patient's functional and cognitive performance, reflected by their ability to carry out activities of daily living (ADL). Two RAI assessments exist: the prospective RAI (RAI‐C) and the retrospective administrative RAI (RAI‐A). The prospective RAI is more sensitive but less specific, while the retrospective RAI is more stringent and based on ACS NSQIP data definitions.

Given the potential importance of frailty as a predictive factor of poor postoperative outcomes after UPPP, it is of interest to identify the best way to quantify frailty to determine the association between frailty and postoperative outcomes after UPPP. The aim of this study is to evaluate and compare the ability of mFI‐5 and RAI in predicting postoperative outcomes after UPPP. We hypothesize that RAI would be better able to predict postoperative outcomes than mFI‐5 after UPPP.

## 2 Methods

### 2.1 Data Source

Patient data were obtained from the American College of Surgeons (ACS) National Surgical Quality Improvement Program (NSQIP) database from 2005 to 2020. The ACS NSQIP is a multi‐institutional and nationally validated clinical database that captures variables for pre‐, intra‐, and postoperative outcomes for patients who underwent surgery at hospitals across the nation. The ACS NSQIP is regarded as highly reliable, as all data points are collected from individual institutions by ACS‐trained surgical clinical reviewers, ensuring precision, accuracy, and reliability. This study was performed under a Health‐Insurance Portability and Accountability Act‐compliant Participant Use Data File and was granted exemption through the Rutgers New Jersey Medical School institutional review board (IRB #: Pro2025002197).

### 2.2 Patient Selection

Adult patients (18 years or older) who underwent uvulopalatopharyngoplasty (UPPP) between January 1, 2005, and December 31, 2020, were identified using the Current Procedural Terminology (CPT) codes listed in Table S1. CPT code selection captures isolated palatal procedures, including contemporary variants such as expansion sphincter pharyngoplasty when coded under palatopharyngoplasty descriptors within NSQIP. Patients were excluded if they underwent concurrent procedures, had missing key data elements, or were older than 90 years (Figure S1). The final cohort (*n* = 2129) was categorized into frailty levels (non‐frail, prefrail, frail, and severely frail) based on RAI and mFI‐5 scoring (Table 1).

**TABLE 1:** Components of frailty indices in uvulopalatopharyngoplasty and associated scoring.

Modified frailty index‐5 (mFI‐5)	Risk analysis index (RAI)
1. Diabetes mellitus (+1)	Age
2. Hypertension (+1)	2 Sex (female = 0, male = +5)
3. Dependent functional status (+1)	3 Cancer diagnosis excluding melanoma (range 13–20)
4. Chronic obstructive pulmonary disease or pneumonia (+1)	4 Unintentional Weight loss of 4.5 kg over 3 months (+5)
5. Congestive heart failure (+1)	5 Renal failure or dialysis (+6)
	6 Congestive heart failure (+4)
	7 Poor appetite (+4)
	8 Shortness of breath at rest or minimal activity (+8)
	9 Residence other than independent living (+8)
	10 Cognitive deterioration
	11 Activities of daily living (ADL) defined as functional status: Mobility (0–4) Eating (0–4) Toilet use (0–4) Hygiene (0–4)

### 2.3 Frailty Index: mFI‐5

The mFI was originally constructed with 11 preoperative variables (mFI‐11). However, in 2014, the ACS NSQIP discontinued mandatory reporting of six of these variables. Therefore, the mFI‐5 is a cumulative count of five specific comorbidities available in the database: functional dependence, diabetes mellitus (DM), chronic obstructive pulmonary disease (COPD), hypertension, and congestive heart failure (CHF) (Table 1). Each comorbidity was assigned one point if present, resulting in a total score ranging from 0 to 5. Patients were stratified into the following groups: nonfrail (0), prefrail (1), frail (2), and severely frail (≥ 3).

### 2.4 Frailty Index: RAI

The respective RAI scoring cutoffs and included variables are based on a previously reported algorithm [26]. Preoperative cognitive decline, which is not collected in NSQIP, was excluded from the RAI calculation in this study. Similarly, a few phased‐out ACS NSQIP variables (e.g., preoperative radiotherapy and chemotherapy) were ignored by convention [19]. Each variable was assigned a predefined weight, and a total score was generated for each patient. Patients were stratified into the following categories: non‐frail (RAI scores ≤ 10), prefrail (RAI scores 11–20), frail (RAI scores 21–30), and severely frail (RAI scores ≥ 31) (Table 1). Given the small number of severely frail patients, estimates for this subgroup were considered exploratory.

### 2.5 Outcome Measures

The primary outcomes were 30‐day mortality and 30‐day postoperative complications, classified using the Clavien–Dindo classification (CDC) system (Table S2) [27]. Specific outcomes of interest included CDC Class II (CD II) complications, CDC Class IV (CD IV) complications, deep surgical site infection (DSSI), extended length of stay (eLOS), and non‐home discharge (NHD). eLOS was defined by NSQIP convention as a hospital stay greater than the 75th percentile for all patients in the cohort. NHD was defined as discharge to a destination other than home.

### 2.6 Statistical Analysis

All statistical analyses were conducted using IBM SPSS Statistics (version 28.0, IBM Corp.) and R statistical software (version 3.5.3, The R Foundation for Statistical Computing). Continuous variables were reported as means with standard deviations or medians with interquartile ranges (IQR), and categorical variables were reported as frequencies and percentages. Univariate and multivariable logistic regression models were generated to assess the independent association between frailty and each postoperative outcome. Multivariate covariates included race, sex, BMI, and operative time. Results were reported as odds ratios (OR) with 95% confidence intervals (CIs). Model discrimination was assessed using receiver operating characteristic (ROC) curves with calculation of the area under the curve (AUC), and differences in AUCs between the RAI and mFI‐5 were compared using the nonparametric DeLong test [28]. For all purposes, *p* < 0.05 was considered statistically significant.

## 3 Results

### 3.1 Characteristics of the Study Population

A total of 2129 patients were included, with a mean age of 56.4 years (SD 15.8). The majority were male (75.1%) and White (86.5%). Hypertension (48.3%), dyspnea (7.9%), and dialysis dependence (5.6%) were the most common comorbidities. Most patients were functionally independent preoperatively (85.1%) and underwent elective procedures (78.7%). By RAI classification, 76.7% of patients were robust, 19.1% prefrail, 3.9% frail, and 0.3% severely frail. By mFI‐5, 62.5% were robust, 26.6% prefrail, 9.8% frail, and 1.1% severely frail. Adverse postoperative events included eLOS in 9.1% of patients, NHD in 6.7%, and 30‐day mortality in 0.2%. On the Clavien–Dindo scale, CD II complications were most common (4.9%), followed by CD IIIb (2.7%) and CD IV (2.2%) (Table 2). Findings for the severely frail cohort should be interpreted cautiously due to limited sample size and wide confidence intervals.

**TABLE 2:** Demographic, clinical, and perioperative characteristics of adult uvulopalatopharyngoplasty patients characterized by frailty screening tool (risk analysis index and modified frailty index‐5, *n* = 2192).

Category	Totals, *n* = 2192	RAI categories					mFI‐5 categories				
		Robust, *n* = 1633	Normal, *n* = 406	Frail, *n* = 84	Severely frail, *n* = 7	*p*	Robust, *n* = 1331	Prefrail, *n* = 566	Frail, *n* = 208	Severely frail, *n* = 24	*p*
*Demographics*											
Sex											
Female, *n* (%)	531 (24.9%)	452 (27.7%)	63 (15.5%)	15 (17.9%)	0 (0%)	**< 0.001**	319 (24%)	147 (26%)	55 (26.4%)	9 (37.5%)	**< 0.001**
Male, *n* (%)	1598 (75.1%)	1180 (72.3%)	343 (84.5%)	69 (82.1%)	7 (100%)	**< 0.001**	1011 (76%)	419 (74%)	153 (73.6%)	15 (62.5%)	**< 0.001**
Race, *n* (%)						**< 0.001**					**< 0.001**
White, *n* (%)	1310 (61.5%)	955 (58.5%)	288 (70.9%)	61 (72.6%)	6 (85.7%)		788 (59.2%)	383 (67.7%)	124 (59.6%)	15 (62.5%)	
Black, *n* (%)	257 (12.1%)	199 (12.2%)	49 (12.1%)	9 (10.7%)	0 (0%)		137 (10.3%)	81 (14.3%)	33 (15.9%)	6 (25%)	
Asian, *n* (%)	66 (3.1%)	56 (3.4%)	9 (2.2%)	1 (1.2%)	0 (0%)		41 (3.1%)	17 (3%)	8 (3.8%)	0 (0%)	
Hispanic, *n* (%)	161 (7.6%)	142 (8.7%)	13 (3.2%)	6 (7.1%)	0 (0%)		115 (8.6%)	28 (4.9%)	18 (8.7%)	0 (0%)	
Other^a^, *n* (%)	335 (15.7%)	281 (17.2%)	47 (11.6%)	7 (8.3%)	1 (14.3%)		250 (18.8%)	57 (10.1%)	25 (12%)	3 (12.5%)	
*Preoperative clinical status*											
Hypertension, *n* (%)	713 (33.5%)	410 (25.1%)	240 (59.1%)	56 (66.7%)	7 (100%)	**< 0.001**	0 (0%)	483 (85.3%)	206 (99%)	24 (100%)	**< 0.001**
CHF, *n* (%)	6 (0.3%)	0 (0%)	5 (1.2%)	1 (1.2%)	0 (0%)	**< 0.001**	0 (0%)	1 (0.2%)	2 (1%)	3 (12.5%)	**< 0.001**
COPD, *n* (%)	79 (3.7%)	25 (1.5%)	42 (10.3%)	11 (13.1%)	1 (14.3%)	**< 0.001**	0 (0%)	25 (4.4%)	34 (16.3%)	20 (83.3%)	**< 0.001**
Dyspnea, *n* (%)	138 (6.5%)	50 (3.1%)	62 (15.3%)	23 (27.4%)	3 (42.9%)	**< 0.001**	45 (3.4%)	48 (8.5%)	33 (15.9%)	12 (50%)	**< 0.001**
Renal failure, *n* (%)	0 (0%)	0 (0%)	0 (0%)	0 (0%)	0 (0%)	**< 0.001**	0 (0%)	0 (0%)	0 (0%)	0 (0%)	**< 0.001**
Dialysis, *n* (%)	6 (0.3%)	3 (0.2%)	3 (0.7%)	0 (0%)	0 (0%)	0.280	0 (0%)	1 (0.2%)	4 (1.9%)	1 (4.2%)	**< 0.001**
Disseminated cancer, *n* (%)	44 (2.1%)	0 (0%)	0 (0%)	39 (46.4%)	5 (71.4%)	**< 0.001**	12 (0.9%)	24 (4.2%)	7 (3.4%)	1 (4.2%)	**< 0.001**
Weight loss, *n* (%)	78 (3.7%)	3 (0.2%)	43 (10.6%)	27 (32.1%)	5 (71.4%)	**< 0.001**	28 (2.1%)	31 (5.5%)	19 (9.1%)	0 (0%)	**< 0.001**
Non‐home origin, *n* (%)	35 (1.6%)	13 (0.8%)	10 (2.5%)	10 (11.9%)	2 (28.6%)	**< 0.001**	9 (0.7%)	11 (1.9%)	10 (4.8%)	5 (20.8%)	**< 0.001**
Functional status, *n* (%)						**< 0.001**					**< 0.001**
Independent, *n* (%)	2080 (97.7%)	1609 (98.5%)	393 (96.8%)	75 (89.3%)	3 (42.9%)	**< 0.001**	1323 (99.4%)	549 (97%)	192 (92.3%)	6 (25%)	**< 0.001**
Partial dependence, *n* (%)	30 (1.4%)	7 (0.4%)	11 (2.7%)	8 (9.5%)	4 (57.1%)	**< 0.001**	0 (0%)	11 (1.9%)	13 (6.3%)	1 (4.2%)	**< 0.001**
Total dependence, *n* (%)	2 (0.1%)					**< 0.001**					**< 0.001**
Elective surgery status, *n* (%)	1909 (89.7%)	1474 (90.3%)	359 (88.4%)	71 (84.5%)	5 (71.4%)	**< 0.001**	1207 (90.7%)	497 (87.8%)	188 (90.4%)	17 (70.8%)	**< 0.001**
*Postoperative outcomes*											
Any wound complication, *n* (%)	126 (5.9%)	54 (3.3%)	58 (14.3%)	13 (15.5%)	1 (14.3%)	**< 0.001**	58 (4.4%)	41 (7.2%)	21 (10.1%)	6 (25%)	**< 0.001**
SSI, *n* (%)	48 (2.3%)	19 (1.2%)	24 (5.9%)	5 (6%)	0 (0%)		21 (1.6%)	16 (2.8%)	7 (3.4%)	4 (16.7%)	
DSSI, *n* (%)	29 (1.4%)	10 (0.6%)	16 (3.9%)	2 (2.4%)	1 (14.3%)		10 (0.8%)	10 (1.8%)	9 (4.3%)	0 (0%)	
OSSI, *n* (%)	27 (1.3%)	16 (1%)	8 (2%)	3 (3.6%)	0 (0%)		15 (1.1%)	8 (1.4%)	3 (1.4%)	1 (4.2%)	
Dehisc., *n* (%)	42 (2%)	16 (1%)	21 (5.2%)	5 (6%)	0 (0%)		21 (1.6%)	13 (2.3%)	5 (2.4%)	3 (12.5%)	
Clavien–Dindo II, *n* (%)	163 (7.7%)	43 (2.6%)	83 (20.4%)	33 (39.3%)	4 (57.1%)	**< 0.001**	59 (4.4%)	67 (11.8%)	30 (14.4%)	7 (29.2%)	**< 0.001**
Clavien–Dindo IIIb, *n* (%)	174 (8.2%)	89 (5.5%)	65 (16%)	18 (21.4%)	2 (28.6%)	**< 0.001**	77 (5.8%)	67 (11.8%)	26 (12.5%)	4 (16.7%)	**< 0.001**
Clavien–Dindo IV, *n* (%)	68 (3.2%)	26 (1.6%)	31 (7.6%)	11 (13.1%)	0 (0%)	**< 0.001**	24 (1.8%)	27 (4.8%)	14 (6.7%)	3 (12.5%)	**< 0.001**
Mortality, *n* (%)	5 (0.2%)	1 (0.1%)	2 (0.5%)	2 (2.4%)	0 (0%)	**< 0.001**	2 (0.2%)	3 (0.5%)	0 (0%)	0 (0%)	0.383
eLOS, *n* (%)	142 (6.7%)	42 (2.6%)	68 (16.7%)	28 (33.3%)	4 (57.1%)	**< 0.001**	48 (3.6%)	60 (10.6%)	25 (12%)	9 (37.5%)	**< 0.001**
Non‐home discharge, *n* (%)	100 (4.7%)	28 (1.7%)	53 (13.1%)	18 (21.4%)	1 (14.3%)	**< 0.001**	33 (2.5%)	41 (7.2%)	19 (9.1%)	7 (29.2%)	**< 0.001**

*Note:* Bolding indicates statistically significant values on chi‐squared analysis with *p* < 0.05.

Abbreviations: CHF, congestive heart failure; Dehisc, wound dehiscence; DSSI, deep incisional site infection; eLOS, extended length of stay; OSSI, organ/space surgical site infection; SSI, superficial incisional site infection.

^a^ Unknown/Native Hawaiian or Other Pacific Islander/American Indian or Alaska Native.

### 3.2 Regression Analysis

For primary outcomes, frailty by RAI was significantly associated with morbidity and mortality (Table 3). Frail patients had higher odds of CD II (OR 6.44, 95% CI 3.39–12.24; *p* < 0.001), CD IIIb (OR 2.45, 95% CI 1.30–4.62; *p* = 0.006), and CD IV (OR 4.58, 95% CI 1.99–10.57; *p* = 0.006) complications. Severely frail patients specifically demonstrated increased odds of CD II (OR 21.26, 95% CI 3.65–123.77; *p* < 0.001) and mortality (OR 46.66, 95% CI 2.77–768.65; *p* = 0.008). For secondary outcomes, frail patients had increased risk of eLOS (OR 6.34, 95% CI 3.40–11.83; *p* < 0.001) and NHD (OR 6.15, 95% CI 3.00–12.60; *p* < 0.001). Severely frail patients had markedly elevated odds of eLOS (OR 29.36, 95% CI 5.64–160.57; *p* < 0.001). Infectious complications were also observed, with severely frail patients having significantly higher odds of deep SSI (OR 17.54, 95% CI 1.74–176.33; *p* = 0.015).

**TABLE 3:** Multivariate analysis of Clavien–Dindo surgical outcomes across frailty cohorts.

Cohort	CD I (OR, 95% CI)	CD II (OR, 95% CI)	CD IIIb (OR, 95% CI)	CD IV (OR, 95% CI)	CD V (OR, 95% CI)
RAI prefrail	2.765 (1.366–5.597)*	2.980 (1.855–4.787)**	1.501 (1.006–2.240)*	2.719 (1.471–5.024)	9.556 (0.637–143.359)
RAI frail	2.296 (0.767–6.871)	6.442 (3.391–12.239)**	2.450 (1.298–4.623)*	4.582 (1.987–10.567)	46.663 (2.768–768.650)*
RAI severely frail	—	21.262 (3.652–123.768)**	5.837 (0.907–37.573)	—	—
mFI prefrail	0.933 (0.451–0.931)	1.516 (0.943–2.438)	1.470 (0.987–2.188)	0.997 (0.537–1.853)	1.089 (0.159–7.460)
mFI frail	1.126 (0.440–2.881)	2.653 (1.443–4.877)*	1.468 (0.866–2.488)	1.404 (0.670–2.939)	—
mFI severely frail	4.230 (1.165–15.357)*	4.636 (1.271–16.835)*	1.491 (0.451–4.924)	1.392 (0.355–5.455)	—

Abbreviations: CD, Clavien–Dindo; mFI, modified frailty index; RAI, risk analysis index.

* *p* < 0.05.

** *p* < 0.001.

The modified frailty index was associated with select complications (Table 4). Frail patients had higher odds of CD II complications (OR 2.65, 95% CI 1.44–4.88; *p* = 0.002). Severely frail patients specifically demonstrated increased odds of CD I (OR 4.23, 95% CI 1.17–15.36; *p* = 0.028) and CD II (OR 4.64, 95% CI 1.27–16.84; *p* = 0.020), but no significant associations were identified with CD IIIb–IV or mortality. For secondary outcomes, severely frail patients had increased risk of eLOS (OR 4.79, 95% CI 1.66–13.81; *p* = 0.004) and NHD (OR 8.22, 95% CI 2.62–25.74; *p* < 0.001), while frail patients also had increased risk of NHD (OR 2.44, 95% CI 1.25–4.73; *p* = 0.009). Severely frail patients demonstrated higher odds of SSI (OR 4.23, 95% CI 1.17–15.36; *p* = 0.028).

**TABLE 4:** Multivariate analysis of postoperative surgical outcomes across frailty cohorts.

Cohort	eLOS	NHD	SSI	DSSI	OSSI	Dehiscence
RAI prefrail	2.910 (1.832–4.623)**	3.852 (2.242–6.617)**	2.765 (1.366–5.597)*	2.734 (1.127–6.632)*	0.645 (0.244–1.632)	2.047 (0.983–4.264)
RAI frail	6.340 (3.397–11.832)**	6.146 (2.998–12.598)**	2.296 (0.767–6.871)	1.494 (0.298–0.7492)	0.967 (0.255–3.666)	2.083 (0.690–6.286)
RAI severely frail	29.358 (5.638–160.565)**	5.237 (0.529–51.860)	—	17.539 (1.744–176.333)*	—	—
mFI prefrail	1.351 (0.841–2.168)	1.491 (0.873–2.546)	0.933 (0.451–1.931)	0.906 (0.346–2.372)	0.773 (0.299–2)	0.832 (0.382–1.812)
mFI frail	1.649 (0.905–3.006)	2.436 (1.254–4.733)*	1.126 (0.440–2.881)	2.299 (0.848–6.236)	0.783 (0.205–2.991)	0.875 (0.300–2.555)
mFI severely frail	4.793 (1.663–13.808)*	8.215 (2.621–25.743)**	4.230 (1.165–15.357)*	—	1.781 (0.198–16.032)	4.195 (0.982–17.902)

Abbreviations: DSSI, deep surgical site infection; eLOS, extended length of stay; mFI, modified frailty index; NHD, non‐home discharge; OSSI, organ space/site infection; RAI, risk analysis index; SSI, superficial site infection.

* *p* < 0.05.

** *p* < 0.001.

### 3.3 ROC Curve, C‐Statistics, and DeLong Test

**FIGURE 1:**
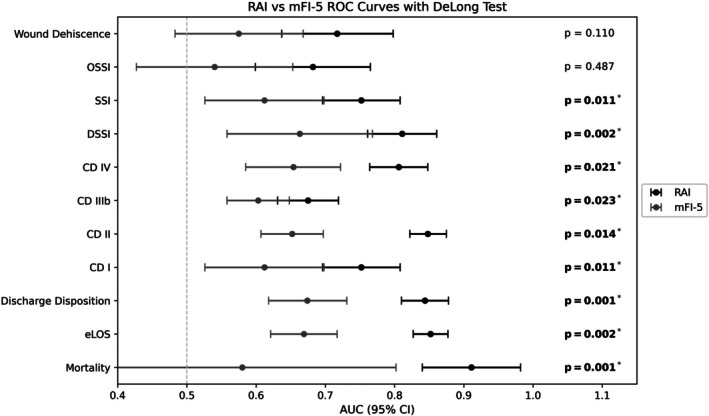
RAI versus mFI‐5 ROC curves with DeLong test for significant difference. Comparison of AUCs (95% CI) for the risk analysis index (RAI) and modified frailty index‐5 (mFI‐5) across postoperative outcomes after uvulopalatopharyngoplasty, with differences assessed using the DeLong test.

**FIGURE 2:**
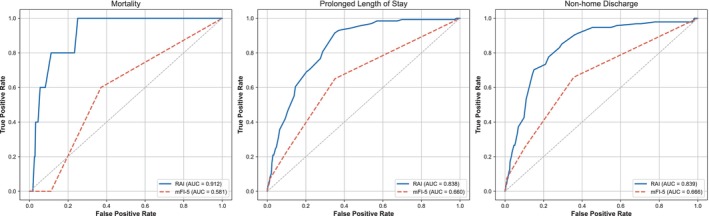
Risk analysis index demonstrates superior discrimination compared to mFI‐5 for key postoperative outcomes after UPPP. ROC curves demonstrating superior discrimination of the risk analysis index compared with mFI‐5 for mortality, extended length of stay, non‐home discharge after uvulopalatopharyngoplasty. [Color figure can be viewed in the online issue, which is available at www.laryngoscope.com]

In ROC analysis (Table 5, Figure 1), RAI had an AUC of 0.911 (95% CI 0.84–0.98) for mortality, compared with 0.580 (95% CI 0.36–0.80) for mFI‐5 (*p* = 0.001). For eLOS, RAI showed an AUC of 0.852 (95% CI 0.83–0.88) compared with 0.669 (95% CI 0.62–0.72) for mFI‐5 (*p* = 0.002), and NHD, RAI had an AUC of 0.844 (95% CI 0.81–0.88) compared with 0.674 (95% CI 0.62–0.73) for mFI‐5 (*p* = 0.001) (Figure 2). RAI also demonstrated higher discrimination than mFI‐5 for CD II complications (0.848 vs. 0.652, *p* = 0.014), deep SSI (0.811 vs. 0.663, p = 0.002), and wound dehiscence (0.717 vs. 0.575, *p* = 0.110).

**TABLE 5:** AUC C‐statistic values from ROC curve analysis of postoperative outcomes.

Cohort	Area	Upper 95% CI	Lower 95% CI
RAI mortality	0.911	0.84	0.982
mFI mortality	0.58	0.357	0.802
RAI eLOS	0.852	0.827	0.877
mFI eLOS	0.669	0.621	0.717
RAI NHD	0.844	0.81	0.878
mFI NHD	0.674	0.618	0.731
RAI CD I	0.752	0.696	0.808
mFI CD I	0.612	0.526	0.698
RAI CD II	0.848	0.822	0.875
mFI CD II	0.652	0.607	0.697
RAI CD IIIb	0.675	0.631	0.719
mFI CD IIIb	0.603	0.558	0.648
RAI CD IV	0.806	0.764	0.848
mFI CD IV	0.654	0.585	0.722
RAI DSSI	0.811	0.761	0.861
mFI DSSI	0.663	0.558	0.768
RAI SSI	0.752	0.696	0.808
mFI SSI	0.612	0.526	0.698
RAI OSSI	0.682	0.599	0.765
mFI OSSI	0.54	0.427	0.653
RAI wound dehiscence	0.717	0.637	0.798
mFI wound dehiscence	0.575	0.483	0.668

Abbreviations: CD, Clavien–Dindo; DSSI, deep surgical site infection; eLOS, extended length of stay; mFI, modified frailty index; NHD, non‐home discharge; OSSI, organ space/site infection; RAI, risk analysis index; SSI, superficial site infection.

## 4 Discussion

In this study, the predictive performance of the RAI and the mFI‐5 was compared across a large cohort of 2129 UPPP patients. Overall, both indices offered predictive value, but the RAI demonstrated stronger discrimination than mFI‐5 for several clinically important outcomes on multivariate analysis [29]. In ROC analysis, RAI showed significantly higher AUCs than mFI‐5 for mortality (0.911 vs. 0.580, *p* = 0.001), eLOS (0.852 vs. 0.669, *p* = 0.002), and NHD (0.844 vs. 0.674, *p* = 0.001). RAI also outperformed mFI‐5 in the prediction of CD II complications (0.848 vs. 0.652, *p* = 0.014) and deep surgical site infection (0.811 vs. 0.663, *p* = 0.002). For wound dehiscence, both indices showed limited discrimination (0.717 vs. 0.575, *p* = 0.110), suggesting neither tool adequately captures risk for this specific outcome. The observed complication rates are consistent with prior large database studies of UPPP, supporting the external validity of our cohort [30, 31, 32]. These findings indicate that, while both indices are associated with adverse events, RAI provided more robust predictive ability across key surgical outcomes. To our knowledge, this is the first study to compare RAI and mFI‐5 in sleep surgery [7].

The magnitude of association in our cohort underscores the clinical salience of frailty [33]. While the absolute rates of the outcomes assessed were relatively low, such effect sizes illustrate how physiologic reserve, rather than chronologic age or comorbidity burden alone, drives recovery potential [34]. This is consistent with prior literature within spine, neurosurgical, head and neck, and thyroid/parathyroid cohorts in which RAI and mFI‐5 cohorts were also compared, yielding similar results and demonstrating consistent effects across various sample sizes [7, 19, 35, 36, 37]. The multidimensional scope of RAI—which incorporates functional status, nutrition, dyspnea, cancer history, and social support—likely explains its broader predictive reach compared with the comorbidity‐based mFI‐5 [38].

Surgical decision‐making for OSA requires balancing operative risk against therapeutic benefit. Frailty screening should therefore function not as the sole decision‐making device but as an additional tool to refine patient selection, guide perioperative planning, and align surgical risk with physiologic reserve. Current preoperative risk stratification for uvulopalatopharyngoplasty (UPPP) relies heavily on anatomic predictors such as Friedman stage, tonsil size, and the apnea–hypopnea index (AHI), which help estimate the likelihood of surgical success [39, 40, 41]. However, these measures do not adequately capture physiologic reserve or vulnerability to adverse outcomes. Against this backdrop, these findings highlight the clinical value of integrating frailty assessment into UPPP preoperative workflows for several reasons. First, frailty screening complements anatomic risk models by identifying patients at higher risk for complications and resource‐intensive recoveries, enabling more comprehensive risk stratification [42, 43, 44]. Second, frailty provides early insight into perioperative risk, allowing surgeons to anticipate resource needs and recovery trajectories. Third, it strengthens shared decision‐making by helping patients and families contextualize individualized perioperative risk alongside anticipated benefit [45].

Critically, frailty becomes clinically actionable when it prompts escalation of care, such as prompting planned admission, tailored Enhanced Recovery After Surgery (ERAS) protocols, and earlier care coordination for at‐risk patients, thereby translating risk prediction into operational decision‐making. Beyond individual care, frailty has been linked to increased costs and readmissions in other surgical populations, suggesting that RAI‐based screening could serve as a system‐level tool for anticipating utilization and optimizing resource allocation [38, 45, 46]. Notably, the RAI has already been operationalized within several surgical specialties through EMR/automated screening workflows, supporting its feasibility as a standardized component of preoperative risk assessment, analogous to ASA classification, in otolaryngology [7, 47].

Both indices have strengths and limitations. The mFI‐5 remains appealing for its simplicity and minimal data requirements. However, aligning with previous studies, mFI‐5 failed to predict severe complications or infection risk [35]. Its widespread adoption within the field is mainly due to the late entry of otolaryngology into frailty research and the rapid adoption of the mFI‐5 as the most established framework [48]. RAI provides greater granularity and predictive accuracy but requires more extensive variable capture and is not yet routinely implemented in otolaryngology practice [36]. Integration into electronic medical records and point‐of‐care calculators may mitigate this barrier [49].

Future research should prospectively validate RAI in sleep surgery populations and directly compare RAI‐C with RAI‐A for UPPP [25]. Developing a hybrid risk calculator that integrates frailty indices with anatomic staging systems (e.g., Friedman stage, Sher criteria) and polysomnography measures may yield a more complete model of surgical candidacy [50]. Additionally, interventional studies should test whether risk‐informed management strategies—such as nutritional optimization, respiratory prehabilitation, or frailty‐tailored ERAS protocols—can improve outcomes [51]. Finally, health‐economic evaluations are needed to determine whether frailty‐based risk stratification reduces costs and unplanned resource utilization. By integrating frailty assessment into contemporary sleep surgery populations, clinicians may move toward a more personalized, multidisciplinary approach to OSA management—aligning patient selection, perioperative care, and resource allocation with individual physiologic reserve [52].

### 4.1 Limitations

This study has several limitations. Its retrospective design precludes causal inference, and reliance on the ACS‐NSQIP database constrains the granularity of available data. Important UPPP‐specific details, including surgical technique, primary versus revision procedures, and baseline OSA severity, were not captured. Similarly, long‐term outcomes such as disease recurrence, CPAP adherence, and quality of life were unavailable. Frailty was assessed using the administrative RAI‐A, which excludes certain domains incorporated in the prospective clinical RAI‐C, such as cognitive decline, potentially underestimating frailty in some patients. The severely frail subgroup was small, limiting estimate precision; therefore, these findings should be considered hypothesis‐generating. Additionally, while NSQIP is nationally validated and risk‐adjusted, coding limitations and missing data may have introduced residual confounding. Despite these constraints, the large national cohort and consistent superiority of RAI over mFI‐5 across multiple outcomes enhance the robustness and generalizability of our findings.

## 5 Conclusion

In this retrospective analysis of over 2000 UPPP patients, the RAI consistently demonstrated superior predictive performance compared to the mFI‐5 across a range of postoperative outcomes, including mortality, serious complications, prolonged hospitalization, and NHD. While mFI‐5 maintained modest predictive value for select endpoints, it failed to identify patients at risk for more severe complications and infection‐related events. These findings support the clinical utility of RAI as a more comprehensive and discriminative tool for frailty assessment in sleep surgery populations. Integrating RAI into preoperative workflows may enhance risk stratification, facilitate targeted perioperative planning, and improve shared decision‐making for patients undergoing UPPP. Future work should focus on prospective validation of RAI in otolaryngology, development of hybrid risk models combining physiologic and anatomic predictors, and evaluation of whether frailty‐informed interventions can meaningfully alter postoperative trajectories. Collectively, these findings support expanding preoperative evaluation beyond anatomic predictors to incorporate physiologic reserve when assessing surgical candidacy in contemporary sleep surgery.

## Funding

The authors have nothing to report.

## Conflicts of Interest

The authors declare no conflicts of interest.

## Supporting information


**Figure S1:** Flowchart of patient selection for uvulopalatopharyngoplasty cohort. Flowchart illustrating patient selection, exclusion criteria, and frailty stratification for the final analytic cohort of patients undergoing isolated uvulopalatopharyngoplasty.

**Table S1:** CPT codes for Uvulopalatopharyngoplasty.
**Table S2:** Clavien–Dindo classification system.

## Data Availability

The data that support the findings of this study are openly available in National Surgical Quality Improvement Program at https://www.facs.org/quality‐programs/data‐and‐registries/acs‐nsqip/.

## References

[1] D. Rana , C. Torrilus , W. Ahmad , N. A. Okam , T. Fatima , and N. Jahan , “Obstructive Sleep Apnea and Cardiovascular Morbidities: A Review Article,” Cureus 12, no. 9 (2020): e10424. 32953361 10.7759/cureus.10424 PMC7494423

[2] S. Fujita , W. Conway , F. Zorick , and T. Roth , “Surgical Correction of Anatomic Abnormalities in Obstructive Sleep Apnea Syndrome: Uvulopalatopharyngoplasty,” Otolaryngology—Head and Neck Surgery 89, no. 6 (1981): 923–934. 6801592 10.1177/019459988108900609

[3] D. Sheen and S. Abdulateef , “Uvulopalatopharyngoplasty,” Oral and Maxillofacial Surgery Clinics of North America 33, no. 2 (2021): 295–303. 33581977 10.1016/j.coms.2021.01.001

[4] J. Sundman , P. Nerfeldt , J. Fehrm , J. Bring , N. Browaldh , and D. Friberg , “Effectiveness of Tonsillectomy vs Modified Uvulopalatopharyngoplasty in Patients With Tonsillar Hypertrophy and Obstructive Sleep Apnea: The TEAMUP Randomized Clinical Trial,” JAMA Otolaryngology—Head & Neck Surgery 148, no. 12 (2022): 1173–1181. 36326742 10.1001/jamaoto.2022.3432 PMC9634593

[5] D. Gozal , H.‐L. Tan , L. Kheirandish‐Gozal , D. Gozal , H.‐L. Tan , and L. Kheirandish‐Gozal , “Treatment of Obstructive Sleep Apnea in Children: Handling the Unknown With Precision,” Journal of Clinical Medicine 9, no. 3 (2020): 888. 32213932 10.3390/jcm9030888 PMC7141493

[6] J. A. Tang , A. M. Salapatas , L. B. Bonzelaar , and M. Friedman , “Long‐Term Incidence of Velopharyngeal Insufficiency and Other Sequelae Following Uvulopalatopharyngoplasty,” Otolaryngology—Head and Neck Surgery 156, no. 4 (2017): 606–610. 28116979 10.1177/0194599816688646

[7] L. Evans , C. Moffatt , K. Niknejad , et al., “Risk Analysis Index Frailty Score as a Predictor of Otolaryngology Surgical Outcomes,” Otolaryngology—Head and Neck Surgery 171, no. 6 (2024): 1728–1735. 38988306 10.1002/ohn.899 PMC11605035

[8] P. P. J. K. R. S. L. Li , “The Importance of In‐Hospital Mortality for Patients Requiring Free Tissue Transfer for Head and Neck Oncology,” British Journal of Oral and Maxillofacial Surgery 51, no. 6 (2013): 508–513. 23369783 10.1016/j.bjoms.2012.10.020

[9] B. Ali , E. H. E. Choi , V. Barlas , T. R. Petersen , N. G. Menon , and N. T. Morrell , “Risk Factors for 30‐Day Mortality After Head and Neck Microsurgical Reconstruction for Cancer: NSQIP Analysis,” OTO Open 5, no. 3 (2021): 2473974X211037257. 10.1177/2473974X211037257 PMC8489772 34616994

[10] N. B. Abt , Y. Xie , S. V. Puram , J. D. Richmon , and M. A. Varvares , “Frailty Index: Intensive Care Unit Complications in Head and Neck Oncologic Regional and Free Flap Reconstruction,” Head & Neck 39, no. 8 (2017): 1578–1585. 28449296 10.1002/hed.24790

[11] N. G. Cuccolo , S. Sparenberg , A. M. S. Ibrahim , D. T. Crystal , L. L. Blankensteijn , and S. J. Lin , “Does Age or Frailty Have More Predictive Effect on Outcomes Following Pedicled Flap Reconstruction? An Analysis of 44,986 Cases,” Journal of Plastic Surgery and Hand Surgery 54, no. 2 (2020): 67–76. 31738641 10.1080/2000656X.2019.1688166

[12] O. Zaslavsky , B. B. Cochrane , H. J. Thompson , N. F. Woods , J. R. Herting , and A. LaCroix , “Frailty: A Review of the First Decade of Research,” Biological Research for Nursing 15, no. 4 (2013): 422–432. 23086382 10.1177/1099800412462866

[13] A. G. Yocky , O. P. Owodunni , E. N. Courville , et al., “The Risk Analysis Index Has Superior Discrimination Compared With the Modified Frailty Index‐5 in Predicting Worse Postoperative Outcomes for the Octogenarian Neurosurgical Patient,” Neurosurgery Practice 4, no. 3 (2023): e00044. 39958794 10.1227/neuprac.0000000000000044 PMC11809970

[14] A. C. Panayi , V. Haug , M. Kauke‐Navarro , S. Foroutanjazi , Y. F. Diehm , and B. Pomahac , “The Modified 5‐Item Frailty Index Is a Predictor of Perioperative Risk in Head and Neck Microvascular Reconstruction: An Analysis of 3795 Cases,” American Journal of Otolaryngology 42, no. 6 (2021): 103121. 34171698 10.1016/j.amjoto.2021.103121

[15] B. T. Seibold , C. Dopke , T. Quan , S. Gill , and Z. Zimmer , “Modified Frailty Index Is a Useful Predictor for Complications Following Total Elbow Arthroplasty in All Populations,” Shoulder & Elbow 18 (2025): 371–378. 40321603 10.1177/17585732251338764 PMC12048401

[16] N. V. Shah , D. J. Kim , N. Patel , et al., “The 5‐Factor Modified Frailty Index (mFI‐5) is Predictive of 30‐Day Postoperative Complications and Readmission in Patients With Adult Spinal Deformity (ASD),” Journal of Clinical Neuroscience 104 (2022): 69–73. 35981462 10.1016/j.jocn.2022.07.020

[17] M. Kuwabara , F. Ikawa , N. Michihata , et al., “The 5‐Factor Modified Frailty Index as a More Useful Associated Factor Than Chronological Age After Unruptured Cerebral Aneurysm Surgery: A Nationwide Registry Study,” Neurosurgery 92, no. 2 (2023): 329–337. 36331212 10.1227/neu.0000000000002203 PMC9815091

[18] L. Huang , Z. Li , M. Jian , et al., “Application of MFI‐5 in Severe Complications and Unfavorable Outcomes After Radical Resection of Colorectal Cancer,” World Journal of Surgical Oncology 21, no. 1 (2023): 307. 37752577 10.1186/s12957-023-03186-4 PMC10521557

[19] C. A. Bowers , S. Varela , M. Conlon , et al., “Comparison of the Risk Analysis Index and the Modified 5‐Factor Frailty Index in Predicting 30‐Day Morbidity and Mortality After Spine Surgery,” Journal of Neurosurgery: Spine 39, no. 1 (2023): 136–145. 37029672 10.3171/2023.2.SPINE221019

[20] M. I. Kim , E. W. Stanton , C. Askinas , J. N. Carey , D. A. Daar , and E. C. Koesters , “The 5‐Factor Modified Frailty Index as a Predictor of Outcomes in Abdominal Wall Reconstruction,” Journal of Plastic, Reconstructive & Aesthetic Surgery 104 (2025): 298–306. 10.1016/j.bjps.2025.03.014 40156949

[21] N. P. Patel , F. Elali , D. Coban , et al., “The 5‐Factor Modified Frailty Index (mFI‐5) Predicts Adverse Outcomes After Elective Anterior Lumbar Interbody Fusion (ALIF),” North American Spine Society Journal 13 (2023): 100189. 36579159 10.1016/j.xnsj.2022.100189 PMC9791584

[22] R. Thommen , S. F. Kazim , K. Rumalla , et al., “Preoperative Frailty Measured by Risk Analysis Index Predicts Complications and Poor Discharge Outcomes After Brain Tumor Resection in a Large Multi‐Center Analysis,” Journal of Neuro‐Oncology 160, no. 2 (2022): 285–297. 36316568 10.1007/s11060-022-04135-z

[23] M. Conlon , R. Thommen , S. F. Kazim , et al., “Risk Analysis Index and Its Recalibrated Version Predict Postoperative Outcomes Better Than 5‐Factor Modified Frailty Index in Traumatic Spinal Injury,” Neurospine 19, no. 4 (2022): 1039–1048. 36597640 10.14245/ns.2244326.163 PMC9816576

[24] M. I. Daniel, I , S. D. Aucoin , and C. van Walraven , “A Bayesian Comparison of Frailty Instruments in Noncardiac Surgery: A Cohort Study,” Anesthesia and Analgesia 133, no. 2 (2021): 366–373. 33264118 10.1213/ANE.0000000000005290

[25] D. E. Hall , S. Arya , K. K. Schmid , et al., “Development and Initial Validation of the Risk Analysis Index for Measuring Frailty in Surgical Populations,” JAMA Surgery 152, no. 2 (2017): 175. 27893030 10.1001/jamasurg.2016.4202 PMC7140150

[26] C. A. Bowers , S. Varela , A. F. Naftchi , et al., “Superior Discrimination of the Risk Analysis Index Compared With the 5‐Item Modified Frailty Index in 30‐Day Outcome Prediction After Anterior Cervical Discectomy and Fusion,” Journal of Neurosurgery. Spine 39, no. 4 (2023): 509–519. 37439459 10.3171/2023.5.SPINE221020

[27] P. A. Clavien , J. Barkun , M. L. de Oliveira , et al., “The Clavien‐Dindo Classification of Surgical Complications: Five‐Year Experience,” Annals of Surgery 250, no. 2 (2009): 187–196. 19638912 10.1097/SLA.0b013e3181b13ca2

[28] E. R. DeLong , D. M. DeLong , and D. L. Clarke‐Pearson , “Comparing the Areas Under Two or More Correlated Receiver Operating Characteristic Curves: A Nonparametric Approach,” Biometrics 44, no. 3 (1988): 837–845. 3203132

[29] H.‐S. Lin , J. N. Watts , N. M. Peel , et al., “Frailty and Post‐Operative Outcomes in Older Surgical Patients: A Systematic Review,” BMC Geriatrics 16, no. 1 (2016): 157. 27580947 10.1186/s12877-016-0329-8 PMC5007853

[30] N. Bhattacharyya , “Revisits and Readmissions Following Ambulatory Uvulopalatopharyngoplasty,” Laryngoscope 125, no. 3 (2015): 754–757. 24706474 10.1002/lary.24706

[31] K. A. Franklin , B. Haglund , S. Axelsson , T. Holmlund , N. Rehnqvist , and M. Rosén , “Frequency of Serious Complications After Surgery for Snoring and Sleep Apnea,” Acta Oto‐Laryngologica 131, no. 3 (2011): 298–302. 21133830 10.3109/00016489.2010.528793

[32] E. J. Kezirian , E. M. Weaver , B. Yueh , et al., “Incidence of Serious Complications After Uvulopalatopharyngoplasty,” Laryngoscope 114, no. 3 (2004): 450–453. 15091217 10.1097/00005537-200403000-00012

[33] R. Shah , K. Attwood , S. Arya , et al., “Association of Frailty With Failure to Rescue After Low‐ and High‐Risk Inpatient Surgery,” JAMA Surgery 153, no. 5 (2018): e180214. 29562073 10.1001/jamasurg.2018.0214 PMC5875343

[34] B. Joseph , V. Pandit , B. Zangbar , et al., “Frailty vs Age in Geriatric Trauma Patient Outcome,” JAMA Surgery 149, no. 8 (2014): 766–772. 24920308 10.1001/jamasurg.2014.296

[35] D. Orgun , C. C. Bay , K. M. Carbullido , A. M. Wieland , B. F. Michelotti , and S. O. Poore , “Inconsistent Associations of Modified Frailty Index‐5 With Adverse Head and Neck Reconstruction Outcomes,” Laryngoscope 135, no. 7 (2025): 2342–2352. 39871415 10.1002/lary.32008 PMC12230903

[36] L. K. Evans , C. Tamamian , D. Delavary , et al., “Risk Analysis Index Versus Modified Frailty Index: Outcomes After Otolaryngologic Surgery,” Laryngoscope 135 (2025): 4655–4663. 40747741 10.1002/lary.32408 PMC12706550

[37] A. Warrier , S. Ranganathan , D. Montgomery , et al., “Risk Analysis Index Outperforms the Modified Frailty Index in Predicting Outcomes in Thyroidectomy and Parathyroidectomy,” Otolaryngology–Head and Neck Surgery 174 (2026): 705–714. 41553036 10.1002/ohn.70125 PMC12948393

[38] D. E. Hall , C. A. Jacobs , K. M. Reitz , et al., “Frailty Screening Using the Risk Analysis Index: A User Guide,” Joint Commission Journal on Quality and Patient Safety 51, no. 3 (2025): 178–191. 39855919 10.1016/j.jcjq.2024.12.005 PMC12370012

[39] E. J. Kezirian , E. M. Weaver , B. Yueh , S. F. Khuri , J. Daley , and W. G. Henderson , “Risk Factors for Serious Complication After Uvulopalatopharyngoplasty,” Archives of Otolaryngology—Head & Neck Surgery 132, no. 10 (2006): 1091–1098. 17043257 10.1001/archotol.132.10.1091

[40] T. Kandasamy , E. D. Wright , J. Fuller , et al., “The Incidence of Early Post‐Operative Complications Following Uvulopalatopharyngoplasty: Identification of Predictive Risk Factors,” Journal of Otolaryngology—Head & Neck Surgery 42, no. 1 (2013): 15. 23570393 10.1186/1916-0216-42-15 PMC3650944

[41] J. H. Choi , S. H. Cho , S.‐N. Kim , et al., “Predicting Outcomes After Uvulopalatopharyngoplasty for Adult Obstructive Sleep Apnea,” Otolaryngology—Head and Neck Surgery 155, no. 6 (2016): 904–913. 27484230 10.1177/0194599816661481

[42] C. Zhao , A. V. Jr , Y. Ma , and R. Capasso , “Insights Into Friedman Stage II and III OSA Patients Through Drug‐ Induced Sleep Endoscopy,” Journal of Thoracic Disease 12, no. 7 (2020): 3663–3672. 32802445 10.21037/jtd-20-1471 PMC7399404

[43] D. Petrongari , F. Ciarelli , P. D. Filippo , et al., “Risk and Protective Factors for Obstructive Sleep Apnea Syndrome Throughout Lifespan: From Pregnancy to Adolescence,” Children 12, no. 2 (2025): 216. 40003319 10.3390/children12020216 PMC11854123

[44] S. Y. Jung , Y. M. Mun , G. M. Lee , et al., “The Effect of Multilevel Surgery for Obstructive Sleep Apnea on Fatigue, Stress and Resilience,” Journal of Clinical Medicine 12, no. 19 (2023): 6282. 37834925 10.3390/jcm12196282 PMC10573978

[45] S. L. C. Arya , R. Brahmbhatt , S. Shafii , et al., “Preoperative Frailty Increases Risk of Nonhome Discharge After Elective Vascular Surgery in Home‐Dwelling Patients,” Annals of Vascular Surgery 35 (2016): 19–29. 27263810 10.1016/j.avsg.2016.01.052

[46] C. Montgomery , H. Stelfox , C. Norris , et al., “Association Between Preoperative Frailty and Outcomes Among Adults Undergoing Cardiac Surgery: A Prospective Cohort Study,” Canadian Medical Association Open Access Journal 9, no. 3 (2021): E777–D787. 10.9778/cmajo.20200034 PMC8313095 34285057

[47] P. R. Varley , J. D. Borrebach , S. Arya , et al., “Clinical Utility of the Risk Analysis Index as a Prospective Frailty Screening Tool Within a Multi‐Practice, Multi‐Hospital Integrated Healthcare System,” Annals of Surgery 274, no. 6 (2021): e1230–e1237. 32118596 10.1097/SLA.0000000000003808

[48] A. Warrier , J. Patel , C. A. Bowers , and R. Gurgel , “In Reference to Inconsistent Associations of Modified Frailty Index‐5 With Adverse Head and Neck Reconstruction Outcomes,” Laryngoscope 136, no. 1 (2025): E3–E4. 40899662 10.1002/lary.70098

[49] A. J. Dicpinigaitis , Y. Khamzina , D. E. Hall , et al., “Adaptation of the Risk Analysis Index for Frailty Assessment Using Diagnostic Codes,” JAMA Network Open 7, no. 5 (2024): e2413166. 38787554 10.1001/jamanetworkopen.2024.13166 PMC11127118

[50] H.‐Y. Li , P.‐C. Wang , L.‐A. Lee , N.‐H. Chen , and T.‐J. Fang , “Prediction of Uvulopalatopharyngoplasty Outcome: Anatomy‐Based Staging System Versus Severity‐Based Staging System,” Sleep 29, no. 12 (2006): 1537–1541. 17252884 10.1093/sleep/29.12.1537

[51] P. F. K. Skořepa , A. Alsuwaylihi , D. O'Connor , C. M. Prado , D. Gomez , and D. N. Lobo , “The Impact of Prehabilitation on Outcomes in Frail and High‐Risk Patients Undergoing Major Abdominal Surgery: A Systematic Review and Meta‐Analysis,” Clinical Nutrition 43, no. 3 (2024): 629–648. 38306891 10.1016/j.clnu.2024.01.020

[52] H. M. K. Wong , D. Qi , B. H. M. Ma , et al., “Multidisciplinary Prehabilitation to Improve Frailty and Functional Capacity in High‐Risk Elective Surgical Patients: A Retrospective Pilot Study,” Perioperative Medicine 13, no. 1 (2024): 6. 38263053 10.1186/s13741-024-00359-x PMC10807111

