# Supplementary material for: Risk Analysis Index Versus mFI‐5 for Predicting Outcomes After Uvulopalatopharyngoplasty
Source: Laryngoscope. 2026 Apr 16;136(9):3815–25. doi: 10.1002/lary.70571 (PMC13460899; doi:10.1002/lary.70571)
Supplement: Supplementary file 1 [file LARY-136-3815-s001.pdf]

Include

- Adult (≥18) Surgical Patients in ACS-NSQIP Database
- UPPP Procedures between 1/1/2005 – 12/31/2020

N = 2,439 initially identified

↓

Exclude:

- Concurrent Procedures
- Missing Key Data Elements
- Age > 90 Years

N excluded = 310

↓

Final Analytic Cohort
Patients undergoing isolated UPPP
N = 2,129

↓

Patient Frailty Stratification (using RAI and mFI-5)

- Non-Frail
- Pre-Frail
- Frail
- Severely Frail
