# Supplementary material for: Risk Analysis Index Versus mFI‐5 for Predicting Outcomes After Uvulopalatopharyngoplasty
Source: Laryngoscope. 2026 Apr 16;136(9):3815–25. doi: 10.1002/lary.70571 (PMC13460899; doi:10.1002/lary.70571)
Supplement: Supplementary file 2 [file LARY-136-3815-s002.docx]

**Supplemental Table 1:** CPT Codes for Uvulopalatopharyngoplasty

| **CPT Code** | **Description** | **Use Case** |
| --- | --- | --- |
| 42145 | Excision and Destruction Procedures on the Palate and Uvula (standard UPPP) | Most common UPPP code |
| 42950 | Repair Procedures on the Pharynx, Adenoids, and Tonsils | UPPP for OSA with lateral wall work |
| 42140 | Excision and Destruction Procedures on the Palate and Uvula | Minor/reduced UPPP |
| 42299 | Other Procedures on the Palate or Uvula | Rare/atypical cases |

*UPPP - Uvulopalatopharyngoplasty*

**Supplemental Table 2:** Clavien-Dindo Classification System ^52^

| **Grade** | **Definition** |
| --- | --- |
| Grade I | Any deviation from the normal postoperative course without the need for pharmacological, surgical, endoscopic, or radiological interventions. Allowed therapeutic measures include antiemetics, antipyretics, analgesics, diuretics, electrolytes, and physiotherapy. Includes wound infections treated at the bedside. |
| Grade II | Complications requiring pharmacologic treatment beyond Grade I medications. Includes blood transfusion and total parenteral nutrition. |
| Grade III | Complications requiring surgical, endoscopic, or radiological intervention. |
| *Grade IIIa* | Intervention without general anesthesia. |
| *Grade IIIb* | Intervention under general anesthesia. |
| Grade IV | Life-threatening complications requiring ICU or management of organ dysfunction (including CNS complications). |
| *Grade IVa* | Single organ dysfunction (e.g., dialysis). |
| *Grade IVb* | Multi-organ dysfunction. |
| Grade V | Death of a patient. |
| *Suffix “d”* | Indicates complications causing disability at discharge requiring follow‑up (e.g., “IIIb‑d”). |
